# Autofluorescence of Amyloids Determined by Enantiomeric Composition of Peptides

**DOI:** 10.1021/acs.jpcb.1c00808

**Published:** 2021-05-19

**Authors:** Manuela Grelich-Mucha, Ana M. Garcia, Vladimir Torbeev, Katarzyna Ożga, Łukasz Berlicki, Joanna Olesiak-Bańska

**Affiliations:** † Advanced Materials Engineering and Modelling Group, Wroclaw University of Science and Technology, Wybrzeze Wyspianskiego 27, 50-370 Wroclaw, Poland; ‡ Institute de Science et d’Ingénierie Supramoléculaires (ISIS), International Center for Frontier Research in Chemistry (icFRC), University of Strasbourg, CNRS (UMR 7006) Strasbourg 67000, France; § Department of Bioorganic Chemistry, Faculty of Chemistry, Wroclaw University of Science and Technology, Wybrzeze Wyspianskiego 27, 50-370 Wroclaw, Poland

## Abstract

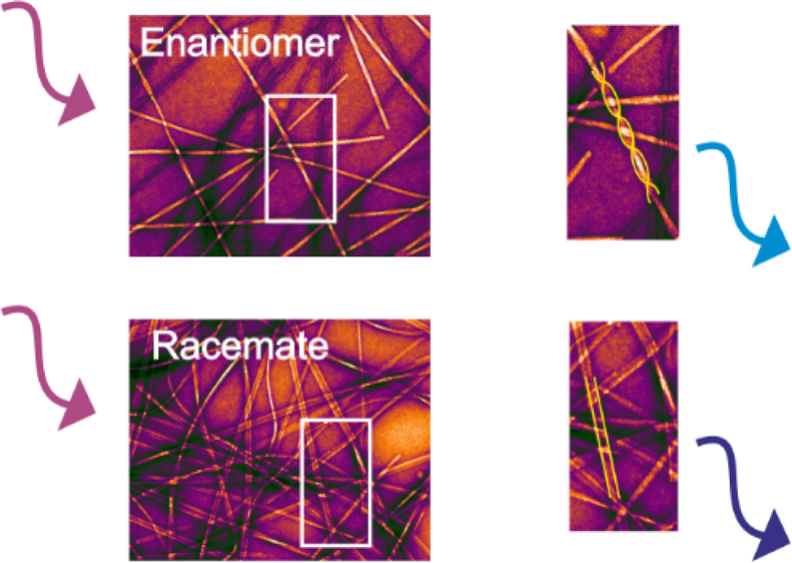

Amyloid fibrils are peptide or protein aggregates possessing a cross-β-sheet structure. They possess intrinsic fluorescence property, which is still not fully understood. Herein, we compare structural and optical properties of fibrils formed from L- and D-enantiomers of the (105–115) fragment of transthyretin (TTR) and from their racemic mixture. Our results show that autofluorescence of fibrils obtained from enantiomers differs from that of fibrils from the racemic mixture. In order to elucidate the origin of observed differences, we analyzed the structure and morphology of fibrils and showed how variations in β-sheet organization influence optical properties of fibrils. We clarified the contribution of aromatic rings and the amyloid backbone to the final blue-green emission of fibrils. This work demonstrates how enantiomeric composition of amino acids allows us to modulate the self-assembly and final morphology of well-defined fibrillar bionanostructures with optical properties controlled by supramolecular organization.

## Introduction

Various peptides and proteins can undergo self-assembly into filamentous structures, commonly known as amyloid fibrils.^1,2^ These species are involved in several neurodegenerative pathologies, including Alzheimer’s or Parkinson’s disease and many other disorders.^3^ Amyloid fibrils share a common cross-β-sheet structural motif, where β-strands are arranged transversely to the long axis of the fibrils.^4,5^

A peculiar property of amyloid fibrils is their intrinsic fluorescence.^6−13^ Fibrils, upon excitation at 350–380 nm, demonstrate fluorescence emission in the visible range, with the maximum centered within 430–450 nm. Importantly, these optical features can also arise in sequences devoid of aromatic amino acid residues,^6,7,13^ what suggests that amyloid autofluorescence falls into a general idea of clustering-triggered luminescence observed for various biomolecules and polymers lacking conventional chromophores.^14,15^ In such systems, through-space conjugation of molecules upon clustering, together with conformational rigidification, results in extended delocalization and observed emission.

The possibility of applying the autofluorescence phenomenon for label-free monitoring of amyloidogenesis^16^ and detection of amyloid deposits in brain^13^ has been demonstrated. However, the mechanism of the effect is still disputable. One possibility is that upon photoexcitation, intermolecular proton transfer occurs along hydrogen bonds connecting N- and C-termini of opposite strands. This process affects ground- and excited-state energies and results in red-shifted emission.^6,12^ It has been also suggested that the observed autofluorescence of amyloid fibrils originates from charge-transfer excitations, involves inter- and intrachain charge delocalization, and hence, depends on the conformation of peptide chains.^17^ Very recently, numerical simulations of simplified amyloidogenic peptides showed that autofluorescence of amyloid structures is governed by the multitude of amide *n*π* states. Excitation is possible due to deplanarization of the amide groups stabilizing *n*π* states, and the blue-green emission occurs because of rigid cross β-sheet arrangement, which prevents nonradiative relaxation pathways.^18^

Despite common features at the atomistic length scale, amyloids present considerable diversity at the molecular level, which gives rise to different morphologies.^19,20^ In order to correlate various morphologies of amyloids with their optical properties, without any variation in the amino acid sequence, here we propose to investigate racemic mixtures of amyloidogenic peptides and to compare their properties with enantiomerically pure amyloids. In their seminal paper in 1953, Pauling and Corey^21^ predicted that enantiomeric peptides with alternating hydrophilic and hydrophobic side chains can co-assemble into pleated β-sheet structures, whereas rippled β-sheets would arise when β-strands of L- and D-peptides alternate. Over the years, a number of articles described differences in morphology achieved by enantiomeric peptides in comparison with their racemic mixtures. For example, it was reported that the complex formed by the two enantiomers of a triblock-type amphiphilic oligopeptide resulted in globular aggregates and impeded nanofiber formation otherwise observed for each single enantiomer.^22^ In contrast, racemic Aβ42 aggregated into amyloid fibrils with similar morphology as for the enantiomerically pure peptides, but more rapidly.^23^ On the other hand, fibrils formed from enantiomers of Aβ40 differ in morphology with those obtained from their racemic mixture.^24^ It was shown that L- or D-peptide sequences with alternating hydrophobic and hydrophilic amino acid residues tend to be organized into pleated β-sheets, whereas their equimolar mixture provides rippled β-sheets in agreement with the original prediction of Pauling and Corey.^25−27^ This aspect has been further validated for Aβ(16–22) consisting of a nonpolar core and polar termini by comparing the morphology of fibrils formed from two enantiomers and the racemic mixture.^28^

Herein, we investigate the structure and optical properties of amyloid fibrils formed by the peptide (105–115) fragment of the human transthyretin (TTR) protein. Under pathological conditions, TTR misfolds inside the body into amyloid fibrils, which contributes to TTR amyloidosis.^29,30^ Full-length TTR, some TTR variants, and two synthetic peptide fragments, TTR(10–20) and TTR(105–115), were reported to form amyloid fibrils *in vitro*.^31−34^ We selected to study the co-assembly of L- and D-enantiomers of TTR(105–115) and their racemic mixture, provided that the structural data for L-TTR(105–115) are available.^32^ We have examined the structural differences between assemblies formed by single enantiomers (both L- and D-peptides) and the racemic mixture using atomic force microscopy (AFM), transmission electron microscopy (TEM), and attenuated total reflectance Fourier-transform infrared (ATR-FTIR) spectroscopy. Furthermore, their intrinsic fluorescence properties were assessed by steady-state spectroscopy techniques. We identified clear differences in the fibril morphology and β-sheet structure, particularly for racemic amyloids. Moreover, we proved their peculiar intrinsic fluorescence properties. Interestingly, fibrils formed from the racemic mixture exhibit blue-shifted excitation and emission spectra compared to fibrils obtained from enantiomerically pure samples. Molecular dynamics (MD) simulations helped to correlate structural differences at the molecular level with the observed properties. Recorded fluorescence excitation–emission maps suggest fluorescence due to noncovalent interactions between aromatic side chains of tyrosines in all samples. The influence of aromatic amino acid arrangement within fibrils and the hydrogen bonding pattern in the β-sheet structure are discussed with respect to observed optical properties.

## Experimental Section

### Peptide Synthesis

L-TTR(105–115) and D-TTR(105–115) (YTIAALLSPYS) were synthesized by the standard 9-fluorenylmethoxycarbonyl (Fmoc) strategy using the Liberty Blue automated microwave peptide synthesizer. Fmoc-Rink Amide AM resin (100–200 mesh, 0.74 mmol/g, 1% DVB) was used, resulting in peptide–^α^amides. Crude-synthesized peptides were purified by semipreparative reverse-phase high-performance liquid chromatography (HPLC). Purity of the fractions was determined using analytical reverse-phase HPLC and liquid chromatography–mass spectrometry (LC–MS).

### Preparation of Amyloid Fibrils

L-TTR(105–115) and D-TTR(105–115), referred to as L-TTR and D-TTR hereafter, were dissolved at concentration 1 mM in 10% (v/v) acetonitrile/water; pH of the solution was adjusted to 2.0 with HCl. Additionally, a racemic mixture of L-TTR and D-TTR was prepared. The samples were incubated during first 2 days at 37 °C in an Eppendorf Thermomixer C and thereafter kept at room temperature (r.t.) for the next 14 days.^33^

### Atomic Force Microscopy

AFM imaging was performed using a Dimension V Veeco AFM instrument in the tapping mode. Characterization of the samples was conducted before and after the incubation period. L-TTR and D-TTR samples were not diluted. The racemic mixture before incubation was also not diluted, whereas for imaging after incubation, the sample was diluted 60-fold. Thereon, 40 μL aliquots of the as-prepared samples were deposited on mica, and after 5 min of the adsorption period, they were rinsed with Milli-Q water and dried. Dimensions of fibrils were estimated based on 50 individual profiles using Nanoscope Software 7.30.

### Transmission Electron Microscopy

TEM micrographs were acquired on a Philips CM12 electron microscope, operating at 80 keV. First, TEM grids (carbon-coated 300 mesh copper TEM grids) were exposed to the UV–ozone cleaner for 5 min. Then, grids were placed coated-side-down for 60 s onto sample drops (20 μL). After this time, the grids were retrieved, washed with deionized water, and stained with 2% (w/v) uranyl acetate for 20 s. Grids were air-dried overnight and then kept under vacuum before analysis. Measurements of fibril dimensions were done using ImageJ software by calculating the average values based on at least 50 individual measurements.

### Absorption and Fluorescence Spectroscopies

For the measurements, both enantiomers were diluted to 166 μM concentration, whereas their racemic mixture, due to strong light scattering, was diluted to 83 μM. Extinction spectra were measured before and after incubation, on a Jasco V-670 spectrophotometer within the range 240–650 nm. Fluorescence excitation and emission spectra were recorded using a FluoroMax-4 spectrofluorimeter (Horiba Jobin Yvon). Excitation spectra were recorded at λ_em_ = 440 nm, whereas emission spectra were recorded at λ_exc_ = 360 nm. Fluorescence excitation–emission intensity maps were recorded in the range λ_exc_ = 270–400 nm and λ_em_ = 330–550 nm, respectively. The excitation and emission slits were set to bring 5 nm resolution.

### ATR-FTIR Spectroscopy

ATR-FTIR spectra were obtained using a Vertex 60v spectrometer. Residual TFA was removed from the samples by treatment with 10 mM HCl and lyophilization (3 cycles). Peptides were dissolved at 1 mM concentration in 10% (v/v) acetonitrile/D_2_O; pH of the solution was adjusted to 2.0 with HCl. The ATR signals from samples were collected 64 times after a background measurement and averaged. The spectra were recorded in the range of 4000–400 cm^–1^ with the resolution equal to 4 cm^–1^.

### Molecular Dynamics Simulations

Initial two-layered parallel β-sheet of L-configuration was taken from the solid-state NMR structure (PDB id: 2m5n). Then, on every second strand, the transformation matrix was applied for 180° rotation and reflection, which resulted in an antiparallel β-sheet composed of alternately occurring L- and D-strands. Eventually, the model was extended by 8 additional strands giving a 24-mer bilayered structure and minimized using the Discovery Studio Smart Minimizer protocol with distance-dependent dielectric as an implicit water model. As a reference, the 24-mer bilayered structure of the parallel β-sheet of L-configuration was also prepared.

To validate the models and their stability, 50 ns long MD (Amber03 force field) was conducted using GROMACS software available on WCSS (Wrocław). Simulation box definition and solvation of the system were performed using gmx editconf and gmx solvate methods using the spc216 water model. The minimization of the system was performed using the steepest descent algorithm with 5000 maximum number of steps and PME electrostatic and converged in 700 steps. Equilibration of the system consisted of two phases: 100 ps (50,000 steps) under the canonical ensemble (*NVT*) and 100 ps (50,000 steps) under the *NPT* ensemble for temperature and pressure stabilization followed by 50,000 ps (2,500,000 steps) production run. All the dynamics were performed under periodic boundary conditions using the leapfrog scheme and PME electrostatics with a 1 nm cut-off at a constant temperature of 300 K and a pressure equal to 1 bar. The trajectory analysis was performed using built-in GROMACS protocols.

### Electrospray Ionization Mass Spectra

The electrospray ionization mass spectra (ESI MS) were obtained using a Waters LC Premier XE mass spectrometer. ESI-MS was conducted for L-TTR(105–115), D-TTR(105–115), and the racemic mixture sample before and after the incubation period. Theoretical simulation of isotopically resolved mass spectra was performed using IsoPro (v.3) software.

## Results and Discussion

**Figure 1:**
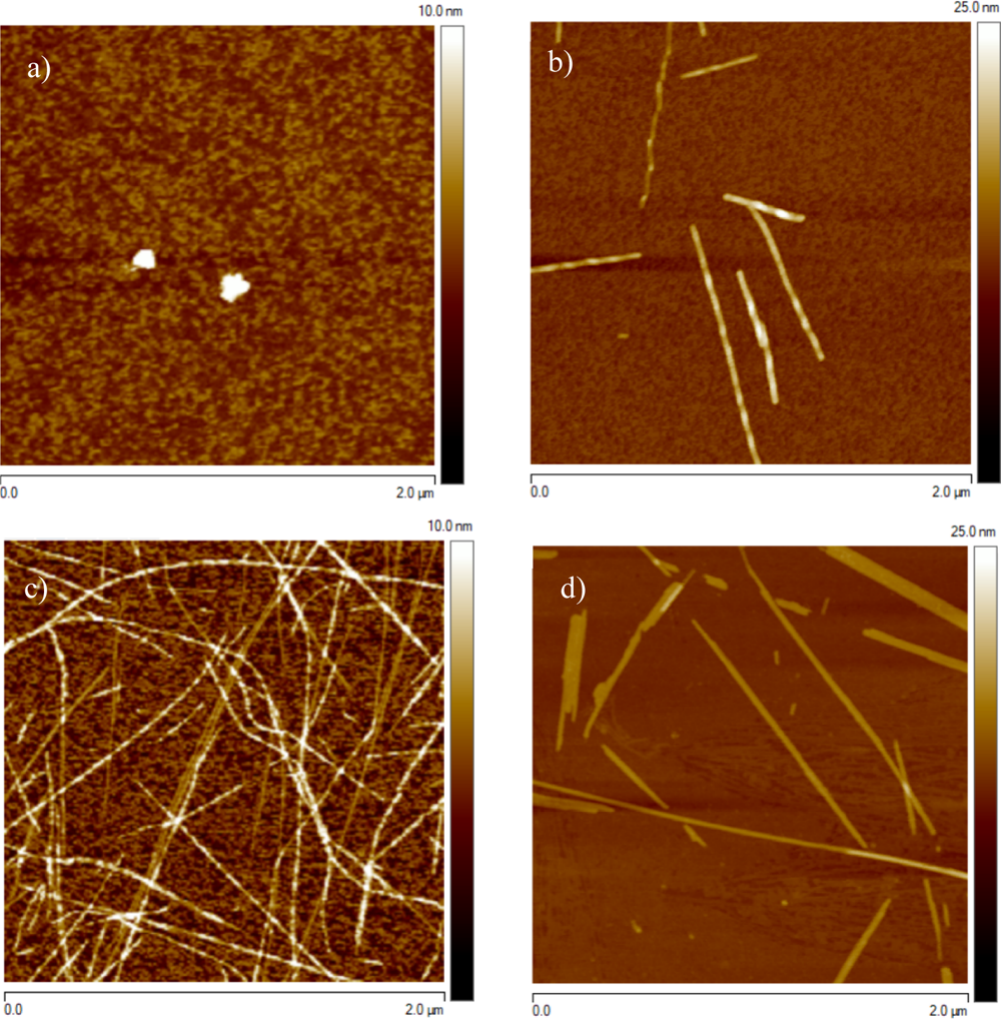
AFM images of L-TTR (a,b) and racemic mixture L/D-TTR (c,d) performed before (a,c) and after (b,d) the formation of mature amyloid fibrils. For images recorded before and after incubation, the scale is set to 10 and 25 nm, respectively.

Enantiopure L-TTR and D-TTR as well as an equimolar racemic mixture of L-TTR and D-TTR were incubated at pH = 2 in order to form amyloids. Previously, it was reported that acidic pH favors amyloid formation of L-TTR.^33^ AFM (Figure 1, Supporting Information Figure S1) and TEM imaging (see Supporting Information Figure S2) revealed formation of amyloid fibrils in all the samples. Before incubation, enantiomeric samples show the formation of globular aggregates (Figure 1a and Supporting Information Figure S1a for L-TTR and D-TTR, respectively), whereas at the same time point in the racemic sample, single protofilaments were already observed (Figure 1c). These observations are in agreement with reported density functional theory simulations, explaining that the formation of racemic rippled β-sheet fibrils is energetically more favorable than that of enantiomerically pure pleated ones.^35,36^ Moreover, the recently accelerated fibrillation process upon mixing of mirror-image peptides was reported.^37^ This also explains why spontaneous resolution from the D/L mixture into enantiopure amyloid fibrils is unfavorable.

After incubation, both enantiomers gave rise to pleated β-sheets forming twisted fibrils (Figure 1b, Supporting Information Figure S2a,b), whereas the racemic mixture yielded tapelike morphology (Figure 1d, Supporting Information Figure S2c). AFM images (Figure 1b, Supporting Information Figure S1b) clearly demonstrate that the pleated β-sheets differ in handedness, as L-TTR resulted in right-handed morphology, whereas D-TTR in left-handed fibrils. The morphology of the fibrils formed from the racemic mixture was totally distinct. The AFM and TEM imaging revealed that the obtained tapelike assemblies have a lower height (4.0 ± 0.9 nm) and are broader with the width reaching a value of 23 ± 5.1 nm (Figure 1d, see Supporting Information Figure S2c). Noteworthy is the fact that for AFM imaging, the L- and D-TTR samples were not diluted, whereas the racemic mixture required 60-fold dilution to obtain the same fibril density. AFM imaging implies that the racemic mixture undergoes accelerated fibril formation compared to the enantiomerically pure peptides generating a larger population of these species. Morphology analysis of structures formed prior to and after incubation demonstrates that mature fibrils obtained from the racemic mixture (Figure 1d and Supporting Information Figure S2c) are approximately 2.5 times wider and 1.5 times higher than structures observed before incubation (Figure 1c). AFM images (Figure 1c) also demonstrate that prior to incubation, besides protofilaments, small peptide aggregates are present, which possibly at later incubation stages get incorporated to form mature amyloid fibrils. We have observed similarities between the L- and D-TTR amyloid fibrils. First of all, AFM analysis (see Supporting Information Figure S3) revealed that L-TTR fibrils are characterized by a periodic twist with the cross-over distance equal to 101 ± 8 nm (see Supporting Information Figure S3b). The average height was calculated to be 7.5 ± 1.7 nm. In order to avoid artefacts resulting from AFM tip-sample convolution, the width was measured using TEM and was equal to 17 ± 3.0 nm. These dimensions are in a close agreement with the data previously reported by Fitzpatrick et al.^38^ Additionally, the width dimensions suggest that the obtained fibrils consist of quadruplets.^38^ Very similar results were noticed for fibrils formed from D-TTR. The cross-over distance was equal to 102 ± 8 nm, and the height and the width were equal 7.4 ± 1.6 and 18 ± 3.4 nm, respectively.

As the enantiomers and the racemic mixture differ in terms of the fibril morphology, we decided to study their intrinsic optical properties. Moreover, chirality-dependent differences in UV absorption spectra can be observed even for pure amino acids, as was for L- and D-enantiomers of lysine.^39^ In our case, extinction spectra (see Supporting Information Figure S4) reveal an absorption band with a maximum at 276 nm, which comes from tyrosine residues.^40^ After the incubation, extinction in all of the samples has higher intensity compared to the values in the corresponding samples before incubation, which confirms the formation of peptide aggregates scattering the incoming light. The exponentially decaying tail in extinction spectra further suggests higher light scattering of the racemic mixture than enantiomers, due to a larger amount of amyloid fibrils in the sample, as confirmed by AFM (Figure 1d) and TEM (see Supporting Information Figure S2c) images.

**Figure 2:**
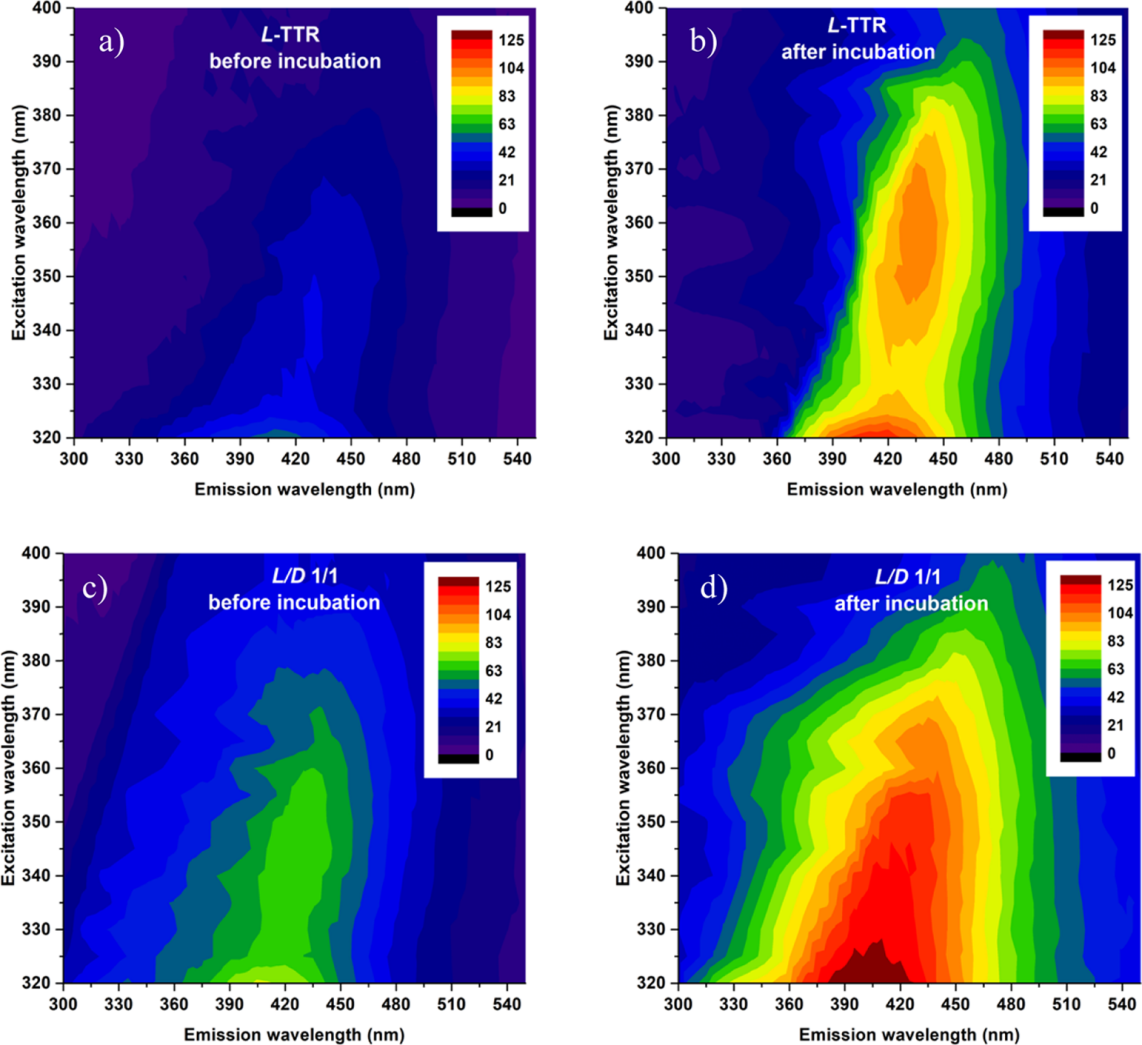
Fluorescence excitation–emission maps with excitation in the range 320–400 nm recorded for the L-TTR peptide fragment and the racemate before (a,c) and after incubation (b,d), respectively. Intensity values were divided by 1000.

**Figure 3:**
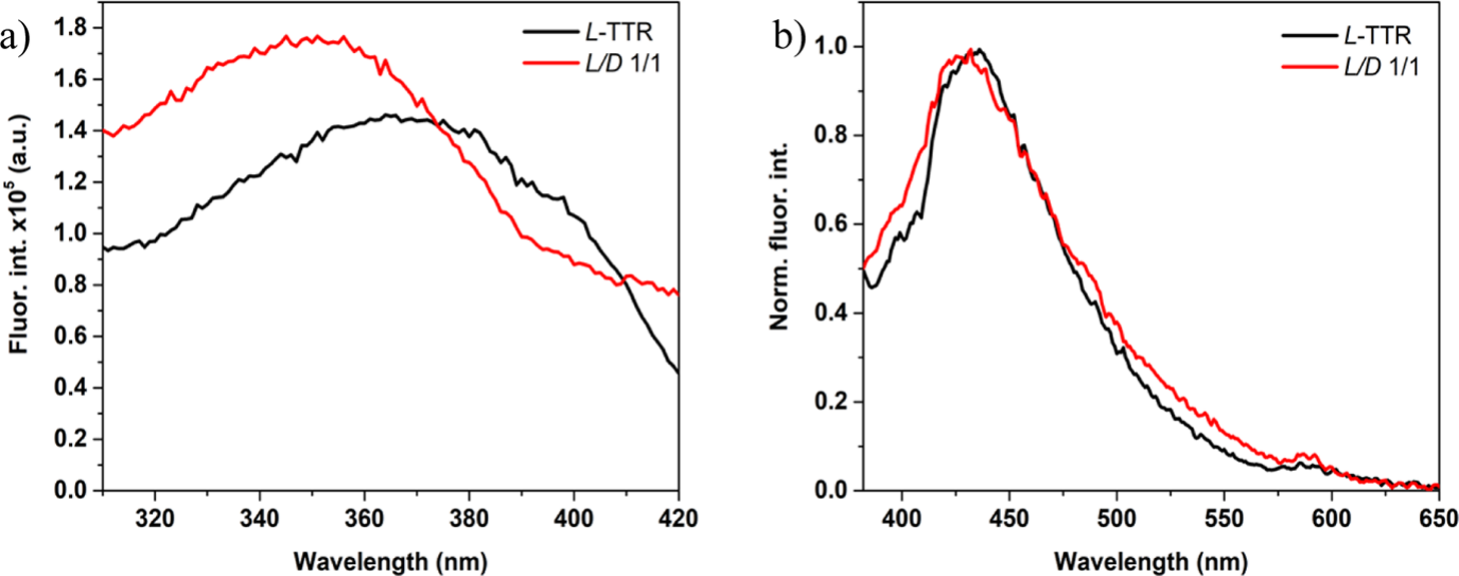
Fluorescence excitation (λ_em_ = 450 nm) (a) and emission (λ_exc_ = 360 nm) (b) spectra recorded for the L-TTR peptide fragment (black lines) and the racemate (red lines) after incubation.

We collected fluorescence excitation–emission intensity maps before and after incubation of the L-TTR peptide and the racemic mixture (Figure 2). Emission spectra of L-TTR recorded in the excitation range 320–400 nm clearly demonstrate that after the incubation, the band with the maximum at λ_em_ ≈ 440 nm appears in a sample. This band excited at ∼360 nm is characteristic of amyloid fibril autofluorescence.^6−8,10,11,13,41−43^ A similar band, with strongly increased intensity after the incubation, is visible in the racemate sample. However, in this case, weak emission is observed already before incubation (Figure 2c), possibly due to the fact that the L/D-TTR 1/1 mixture aggregates and forms fibrils rapidly, already before incubation, as is shown in Figure 1c,d. In order to compare the exact positions of excitation and emission bands of fully grown fibrils, we measured higher resolution spectra of both samples (Figure 3). The fluorescence emission band is slightly blue-shifted in the racemic mixture, with amyloid autofluorescence maximum at 436 and 431 nm, for the enantiomer and the racemic mixture, respectively (Figure 3b, see Supporting Information Figure S5b). Even stronger blue shift is observed in the excitation spectrum, where the enantiomers and the racemic mixture present excitation maxima at 365 and 350 nm, respectively (Figure 3a, see Supporting Information Figure S5a). The excitation (λ_em_ = 450 nm) and emission (λ_exc_ = 360 nm) spectra recorded for the D-form overlay with the ones obtained for the L-form (see Supporting Information Figure S5), which evidence the same spectroscopic features for both enantiomers. The spectral differences in excitation and emission suggest that the local environment of components responsible for amyloid autofluorescence is different in amyloid fibrils of the enantiopure samples and the racemate.

In the excitation–emission maps, both samples present a strong emission at ∼400 nm under excitation <330 nm; thus, we collected additional excitation–emission maps with excitation in the range 270–320 nm (Supporting Information Figure S6). Apart from the Tyr fluorescence for λ_em_ < 350 nm, we observed an additional emission band in the “blue” spectral range, with the maximum λ_em_ ≈ 405 nm. The maximum of the excitation band is located at 292 nm in the case of enantiomeric samples, but in the case of the racemic mixture before incubation, it is located at 290 nm, and after incubation, it is blue-shifted to 286 nm. Interestingly, the emission intensity is significantly lower for the racemate and decreases after incubation (Figure S6d), contrary to the enantiomer sample, where the emission at λ_em_ ≈ 405 nm is stronger after incubation (Figure S6b).

It is described that upon excitation at either 315 nm (alkaline conditions) or 284 nm (acidic conditions), emission is observed in the range 400–420 nm due to the presence of dityrosine (diTyr) residues.^44−47^ In general, the formation of diTyr is promoted by oxygen free radicals, nitrogen dioxide, or hydroxyl radicals.^47,48^ However, it is also possible that upon UV irradiation, tyrosyl radicals are formed and further lead to the formation of diTyr.^46,47^ In order to verify whether in investigated samples diTyr cross-links form or the observed emission results from noncovalent interactions between Tyr residues, we analyzed the samples using electrospray ionization mass spectrometry (see Supporting Information Figure S7). As in ESI-MS signals from ion clusters may occur, we also performed theoretical simulations of masses to unambiguously assign the corresponding peaks in the experimental MS spectra. The MS spectra evidenced the formation of ion clusters (*m*/*z* ≈ 2395 and *m*/*z* ≈ 1797) corresponding to M2 + H^+^ and M3 + 2H^+^ species, respectively, and showed no evidence of covalently linked diTyr species. Therefore, the observed emission at λ_max_ ≈ 405 nm should originate from noncovalent π-stacking interactions between tyrosine residues.

**Figure 4:**
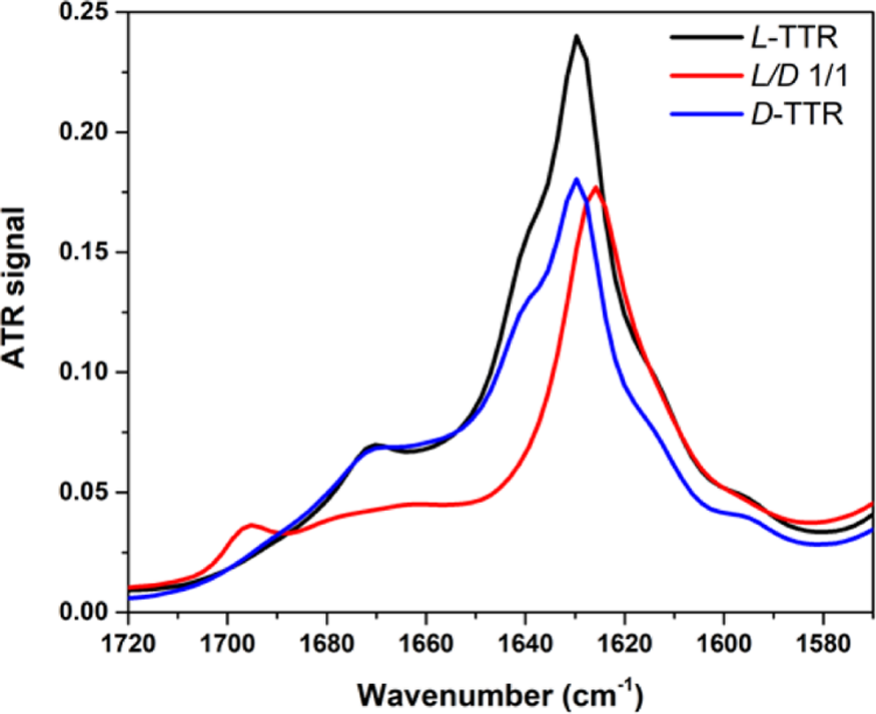
ATR-FTIR spectra of amyloid fibrils formed from L-TTR (black), D-TTR (blue), and the racemic mixture (red). The peak at 1695 cm^–1^ for L/D-TTR reveals the antiparallel β-sheet arrangement, whereas at 1670 cm^–1^ for L- or D-TTR parallel β-sheets.

As suggested by AFM and TEM imaging, the fibrils grown in the enantiopure and racemic mixtures present significantly different morphology. In order to gain an insight into the detailed structure of fibrils, we performed ATR-FTIR measurements. The presence of a β-sheet structure was confirmed in the samples (Figure 4). ATR-FTIR spectra obtained before incubation demonstrate that the racemic mixture, unlike L- and D-TTR, forms a β-sheet structure directly after sample preparation (see Supporting Information Figure S8). They confirm the results observed by AFM and TEM microscopies, that is, the higher tendency toward aggregation of the racemic mixture in comparison to single enantiomers. Comparison of FTIR spectra of the samples recorded after the incubation period (Figure 4) reveals the presence of a clear band in the amide-I region (1615–1630 cm^–1^), which confirms the secondary β-sheet structure in all of the samples. However, there is a significant difference between the racemic mixture (red) and L- or D-TTR (black and blue, respectively) in the exact position of the band: for the racemic mixture, it is located at 1626 cm^–1^, whereas for L- and D-TTR, it is located at 1629 cm^–1^. The lower intensity of the peak at 1629 cm^–1^ recorded for the D-form suggests the lower content of β-sheet structures in the sample, reflected also in AFM and TEM images (Figure 1 and Supporting Information Figures S1 and S2). The shift to lower values for the racemic mixture suggests the increased rigidity of β-sheets.^49,50^ Second, for the racemic mixture, a peak at 1695 cm^–1^ is observed, whereas for L- and D-TTR, the second major peak is at 1670 cm^–1^. It indicates the formation of antiparallel fibrils for the racemic mixture, whereas the fibrils of enantiopure L- and D-TTR form parallel β-sheets. Indeed, a broad shoulder at ∼1660 cm^–1^ suggests the presence of additional disordered features in the racemate.^49,51^ Therefore, we cannot assume that in racemate only the antiparallel β-sheet alignment is present. These findings are in contrast with the investigation of pure enantiomers and equimolar mixtures of amyloid-β,^16−22^ where both pleated β-sheets of pure enantiomers and rippled β-sheets of L/D-Aβ(16–22) consisted of antiparallel β-sheets.^28^

**Figure 5:**
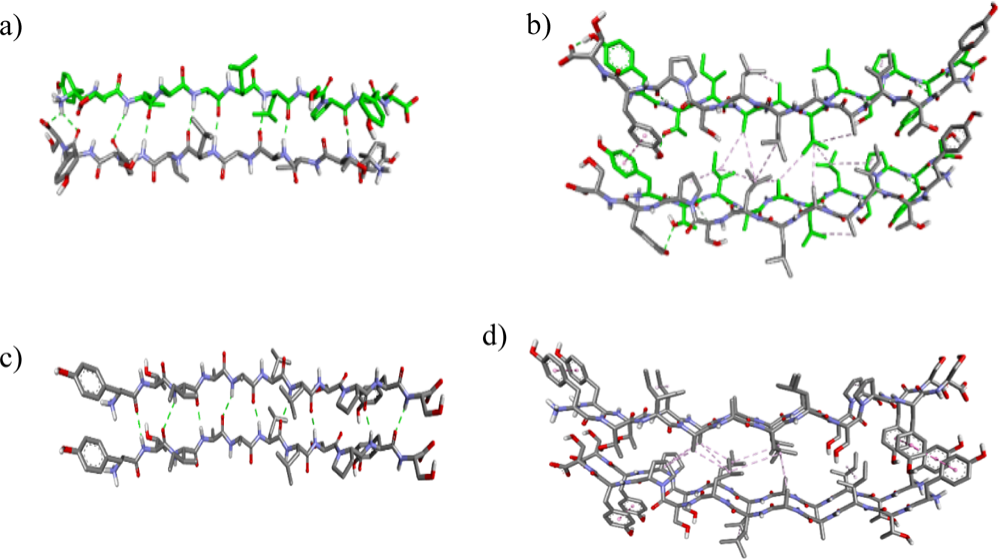
Minimized and equilibrated model of the antiparallel L/D β-sheet and its comparison to the corresponding all-L structure. A pair of antiparallel L and D strands, where hydrogen bonds are represented by a green dotted line. (a) Four-strand section from the side view and (b) hydrophobic interaction between strands belonging to two different layers are represented by a dotted pink line. D-strands are marked in green. A pair of parallel L-TTR(105–115) strands (c) and hydrophobic interactions between L-peptide strands from two different layers (d).

Jaroniec et al.^32^ had previously reported the atomic resolution structure of L-TTR(105–115) amyloids using solid-state NMR. A parallel, in-register organization of β-strands within the fibrils was deduced. The D-TTR(105–115) is the mirror image of L-TTR(105–115) and should also form a parallel β-sheet structure. The racemic mixture of TTR(105–115), as it is evidenced by ATR-FTIR spectra (Figure 4), possesses antiparallel β-sheets, with possible alternate positioning of β-strands from L- and D-TTR in the fibril. Therefore, the enantiomers and the racemic mixture have different hydrogen bonding networks, stabilizing the secondary structure of amyloid fibrils. We have performed MD simulations to get an insight into the structural organization of the racemic mixture (Figure 5a,b) and compared it with the L-TTR(105–115) structure (Figure 5c,d). The proposed structural arrangement of racemic TTR(105–115) was found to be highly stable during MD simulation, as confirmed by virtually invariant values of potential energy, radius of gyration, and distances between atoms of opposite corners of the overall structure during the production run (Supporting Information Figure S9). The stability of this structure is related to a well-defined network of hydrogen bonds (Figure 5a) and well-packed hydrophobic core (Figure 5b). Edge-to-face π–π stacking interactions between side chains of Tyr114 (from L-TTR) to Tyr105 (from D-TTR) as well as Tyr105 (from L-TTR) to Tyr114 (from D-TTR) are present, presumably stabilizing the structure of racemic amyloids. Moreover, it is worth to note that face-to-face interactions of Tyr residue side chains spanning along the axis of the fibril observed for L-TTR(105–115) are absent in the case of the racemic peptide structure due to the alternation of direction of neighboring chains [compare Figure 5b,d; model structures representing single layers composed of alternating L- and D-strands in the racemic mixture and of only L-strands in L-TTR(105–115) are presented in Supporting Information Figure S11]. The alternation of L- and D-peptide chains in the antiparallel β-sheet structure of racemic amyloids of L/D-TTR(105–115) likely explains the lack of twist in the observed flat morphology of racemic amyloids, in contrast to enantiomerically pure amyloids with a noticeable twist.^52^

The fluorescence excitation–emission maps (see Supporting Information Figure S6) demonstrate high intensity emission resulting from noncovalent interactions between tyrosines (λ_exc_ ≈ 290 nm and λ_em_ ≈ 405 nm). It can be noticed that the fluorescence emission at 405 nm in enantiomers increases after the formation of fibrils, whereas in the racemate, a significant decrease of this emission is observed after incubation. These results correlate with the structure of fibrils obtained with MD simulations (Figure 5b,d and Supporting Information Figure S11). In pure enantiomers, the Tyr residues form a continuous π–π stacking chain (Figure 5d), whereas in the racemic mixture, an interaction is restricted to two adjacent Tyr side chains (Figure 5b).

Overall, the emission >400 nm of the TTR enantiomer and the racemate consists of two bands, which depend on β-sheet structure organization of fibrils: the band at ∼435 nm, excited in the 315–410 nm range, which is amyloid-specific autofluorescence and the band at λ_exc_ ≈ 290 nm and λ_em_ ≈ 405 nm, which corresponds to a π–π stacking chain of Tyr residues. The former band is present only when fibrils are formed, whereas the latter band is present regardless of the fibril formation. For enantiomers before incubation, no β-sheet structure was confirmed in ATR-FTIR spectra (see Supporting Information Figure S8a,b) and no amyloid fibrils were observed during AFM imaging (see Figure 1a, Supporting Information Figure S1a). Thus, amyloid-specific fluorescence at ∼435 nm is hardly visible (Figure 3a). A band of amyloid–characteristic transitions at ∼435 nm is clearly visible in all samples after incubation and in the racemate before incubation, where the presence of fibrillar aggregates, with a well-defined β-sheet structure, was confirmed with AFM, TEM, and ATR-FTIR experiments (Figures 1, 4, and Supporting Information Figure S2). These observations support the idea that highly ordered β-strands hinder nonradiative relaxation pathways and allow for observation of amyloid-specific autofluorescence, as was recently investigated by Grisanti and colleagues.^18^ They used *ab initio* calculations and dynamics simulations for modeling double and triple β-strand structures to show the changes in optical properties upon peptide aggregation. They found that in model peptide sequences lacking any aromatic rings and side groups, distortion of amide groups strongly stabilizes *n*π* states and is responsible for excitation bands >300 nm. Amide groups in fibrils are involved in strong hydrogen bonding interactions, with different patterns in the case of parallel and antiparallel organization of β-strands (Figure 5a,c). Thus, in our TTR samples, differences in the H-bond pattern between fibrils may result in observed shifts in a position of amyloid-specific emission bands at ∼435 nm and, even more distinctly, in excitation bands (with maxima at 365 and 350 nm for enantiomers and the racemic mixture, respectively). It is worth noting that in our case, the peptides were synthesized as C-terminal amides, and thus, the contribution to the observed autofluorescence due to intramolecular proton transfer between N- and C- termini, as suggested in some papers,^6,12^ can be excluded. However, we cannot exclude possible short-range charge transfer excitations in the vicinity of charged N-termini, similar to the ones reported by Hassanali and co-workers.^17^

## Conclusions

In summary, we have performed detailed analysis of the structure and optical properties of fibrils formed from peptide fragments L-, D-TTR(105–115), and their racemic mixture. AFM and TEM imaging carried out after the incubation period revealed different morphologies of fibrils: twisted and tapelike fibrils formed by enantiomers and the racemic mixture, respectively. ATR-FTIR measurements evidenced the presence of the secondary β-sheet structure with the parallel β-strand arrangement for L- or D-TTR(105–115) enantiomers and antiparallel arrangement for the racemic mixture. In the racemic mixture, some heterogeneous structures were also present. Performed MD simulations indicated a very stable structure for the racemic TTR(105–115), with the well-defined hydrogen bonding network and well-packed hydrophobic core. Moreover, the racemate presented disrupted long-range π–π stacking between Tyr residues of neighboring peptide chains and different hydrogen bonding patterns in comparison with the structure of a pure enantiomer fibril.

Our studies reveal that these differences found in the fibril structure and morphology result in distinct optical properties for enantiomers and the racemate. Both enantiomers and the racemate exhibited intrinsic fluorescence in the visible range of wavelengths (λ_em_ > 400 nm), similar to autofluorescence described for other amyloids. When fully developed fibrils are formed, an excitation band in the wavelength range of ∼360 nm appears in all samples, but in the case of the racemic mixture, both excitation and emission bands are blue-shifted. The possible explanation of the spectral shift is the differences in the β-sheet structure and the resulting H-bond network organization between enantiomers and the racemate. Moreover, analysis of TTR fluorescence revealed that all samples exhibit fluorescence at λ_em_ ≈ 407 nm, excited at ∼290 nm, due to noncovalent π–π stacking between tyrosine residues. As it is visible before fibril formation, this fluorescence is not amyloid-specific, but also in this case, the final intensity and position of the bands depend on the structure of a fibril being different in enantiomers and the racemate samples.

Overall, we proved that the enantiomeric composition of TTR(105–115) peptides determines the final morphology of amyloid fibrils and their intrinsic autofluorescence in the visible range (>400 nm). Autofluorescence position and intensity are correlated with the structure of a fibril, and therefore, it can serve as a valuable source of information about the internal structure of amyloid fibrils.

## Supporting Information Available

Detailed information regarding AFM and TEM images, extinction spectra recorded before and after the incubation period, excitation and emission spectra recorded for both enantiomers and the racemate after incubation, fluorescence excitation–emission intensity maps in the excitation range of 270–320 nm recorded for L-TTR and the racemate (L/D 1/1) before and after incubation, ESI-MS spectra confirming the absence of covalent Tyr cross-links, ATR-FTIR spectra of all samples recorded before and after incubation, potential energy during minimization of MD simulation of L/D- and L-TTR fibrils, potential energy during production run, radius of gyration, the distance between the opposite corner atoms, and the model structure representing one layer composed of alternating L- and D-strands in the racemic mixture and of adjacent L-strands in the L-TTR(105–115) enantiomer (PDF)

## Abbreviations

TTR: transthyretin
TTR(105–115): transthyretin 105–115 peptide fragment
AFM: atomic force microscopy
TEM: transmission electron microscopy
ATR-FTIR spectroscopy: attenuated total reflectance Fourier transform infrared spectroscopy
MD: molecular dynamics
Fmoc strategy: 9-fluorenylmethoxycarbonyl strategy
HPLC: high-performance liquid chromatography
LC–MS: liquid chromatography–mass spectrometry
r.t.: room temperature
ESI MS: electrospray ionization mass spectra
Tyr: tyrosine
diTyr: dityrosine
