# Supplementary material for: Autofluorescence of Amyloids Determined by Enantiomeric Composition of Peptides
Source: J Phys Chem B. 2021 May 19;125(21):5502–10. doi: 10.1021/acs.jpcb.1c00808 (PMC8182742; doi:10.1021/acs.jpcb.1c00808)
Supplement: Supplementary file 1 — jp1c00808_si_001.pdf [file jp1c00808_si_001.pdf]

Autofluorescence of Amyloids Determined by Enantiomeric Composition of Peptides

Manuela Grelich-Mucha [a], Ana M. Garcia [b], Vladimir Torbeev [b], Katarzyna Ożga [c], Łukasz Berlicki [c], Joanna Olesiak-Bańska [a]*

[a] Advanced Materials Engineering and Modelling Group, Wroclaw University of Science and Technology, Wybrzeze Wyspianskiego 27, 50-370 Wroclaw, Poland.

[b] Institut de Science et d'Ingénierie Supramoléculaires (ISIS), International Center for Frontier Research in Chemistry (icFRC), University of Strasbourg, CNRS (UMR 7006), Strasbourg 67000, France.

[c] Department of Bioorganic Chemistry, Faculty of Chemistry, Wroclaw University of Science and Technology, Wybrzeze Wyspianskiego 27, 50-370 Wroclaw, Poland.

Corresponding author's e-mail: joanna.olesiak@pwr.edu.pl

EXPERIMENTAL SECTION

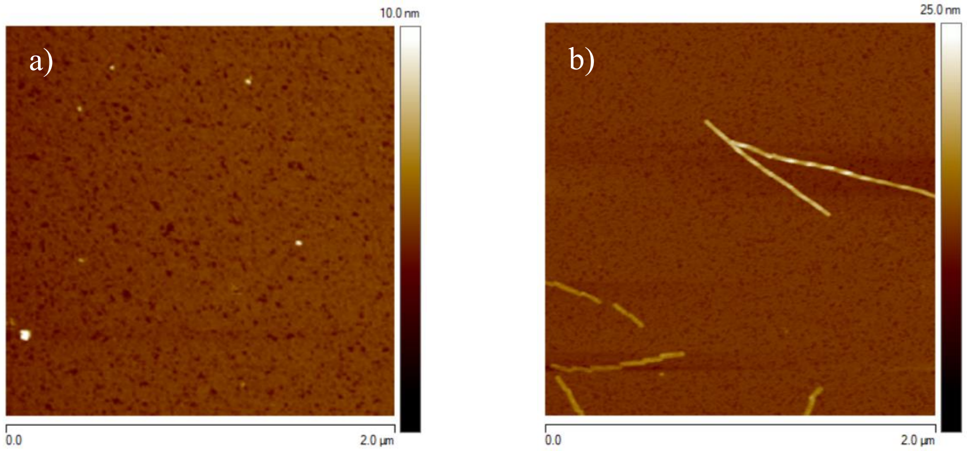

Figure S1. AFM images of *D*-TTR sample investigated prior (a) and after the incubation (b). For the images recorded before and after incubation, scale is set to 10 and 25 nm, respectively.

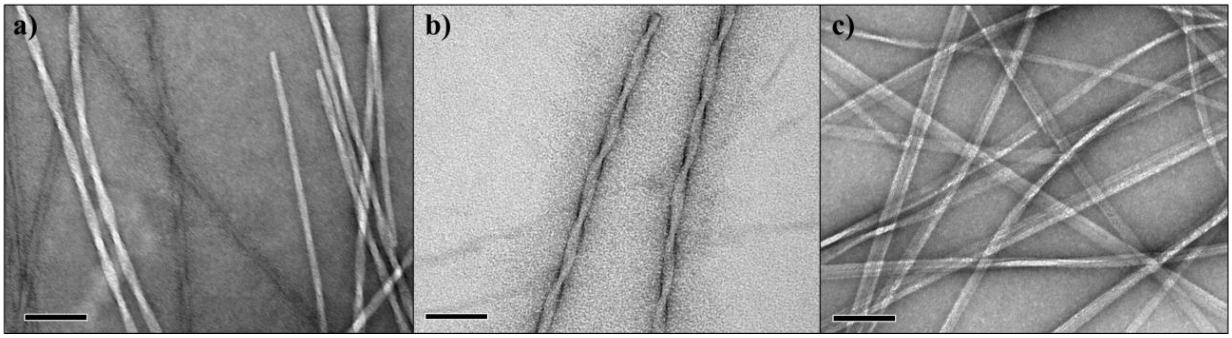

Figure S2. TEM micrographs of *L*-TTR (a), *D*-TTR (b) and *L*/*D*-TTR (c) incubated for 14 days in MeCN/H_2O (1:9) at pH 2. Scale bars = 200 nm.

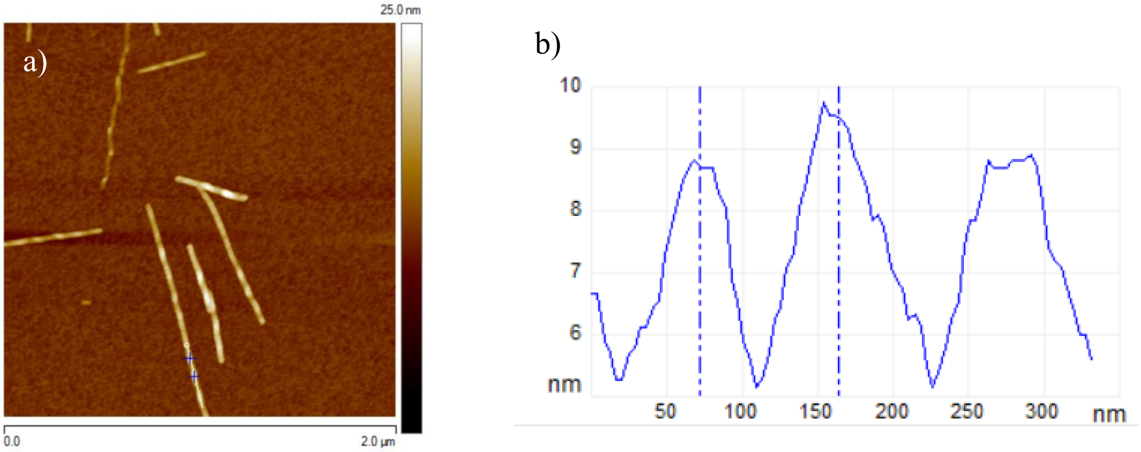

Figure S3. AFM image of fibrils formed from *L*-TTR (a) and the corresponding height profile (b) revealing regular cross-over distance among the fibrils' length (cross-over distance equal 93 nm, as denoted with vertical lines).

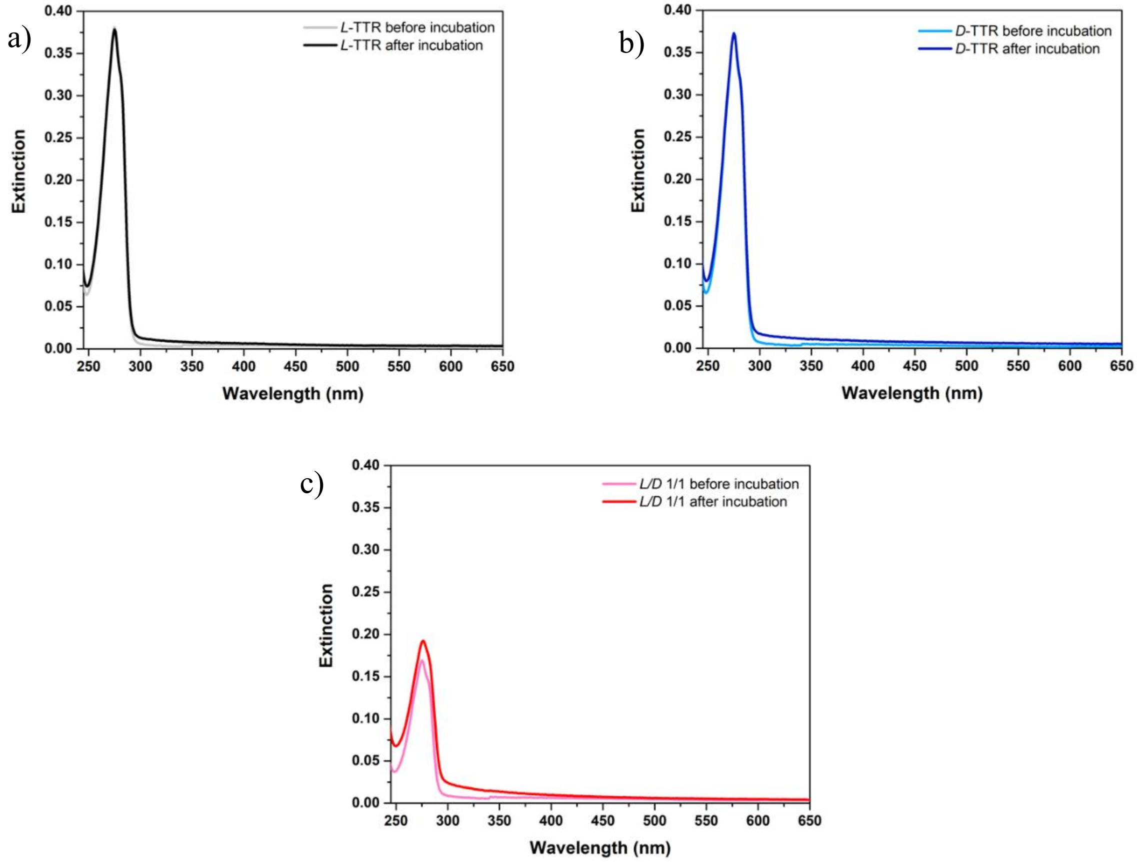

Figure S4. Extinction spectra of samples recorded before and after incubation: *L*-TTR (grey and black) (a), *D*-TTR (light blue and blue) (b), racemic mixture *L/D* 1/1 (rose and red) (c), respectively.

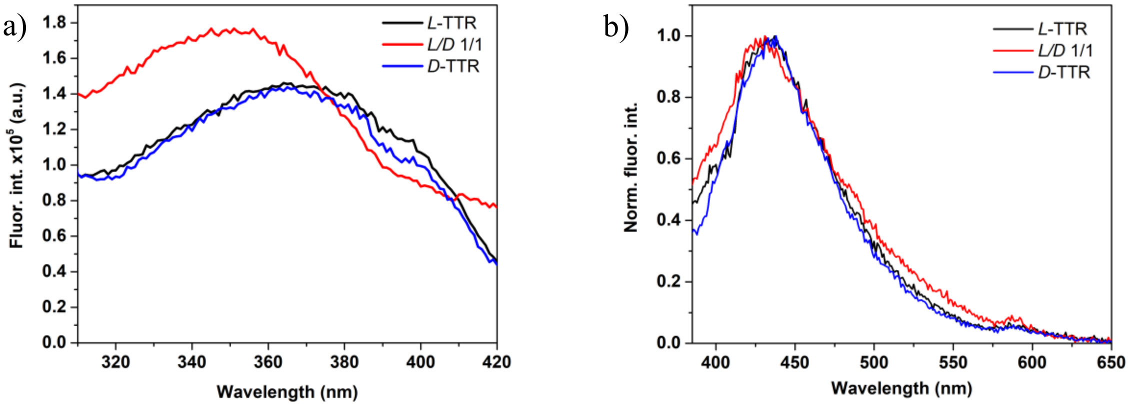

Figure S5. Fluorescence excitation (λem = 450 nm) (a) and emission (λexc = 360 nm) (b) spectra recorded for *L*-TTR (black), the racemate (red) and *D*-TTR (blue) after incubation.

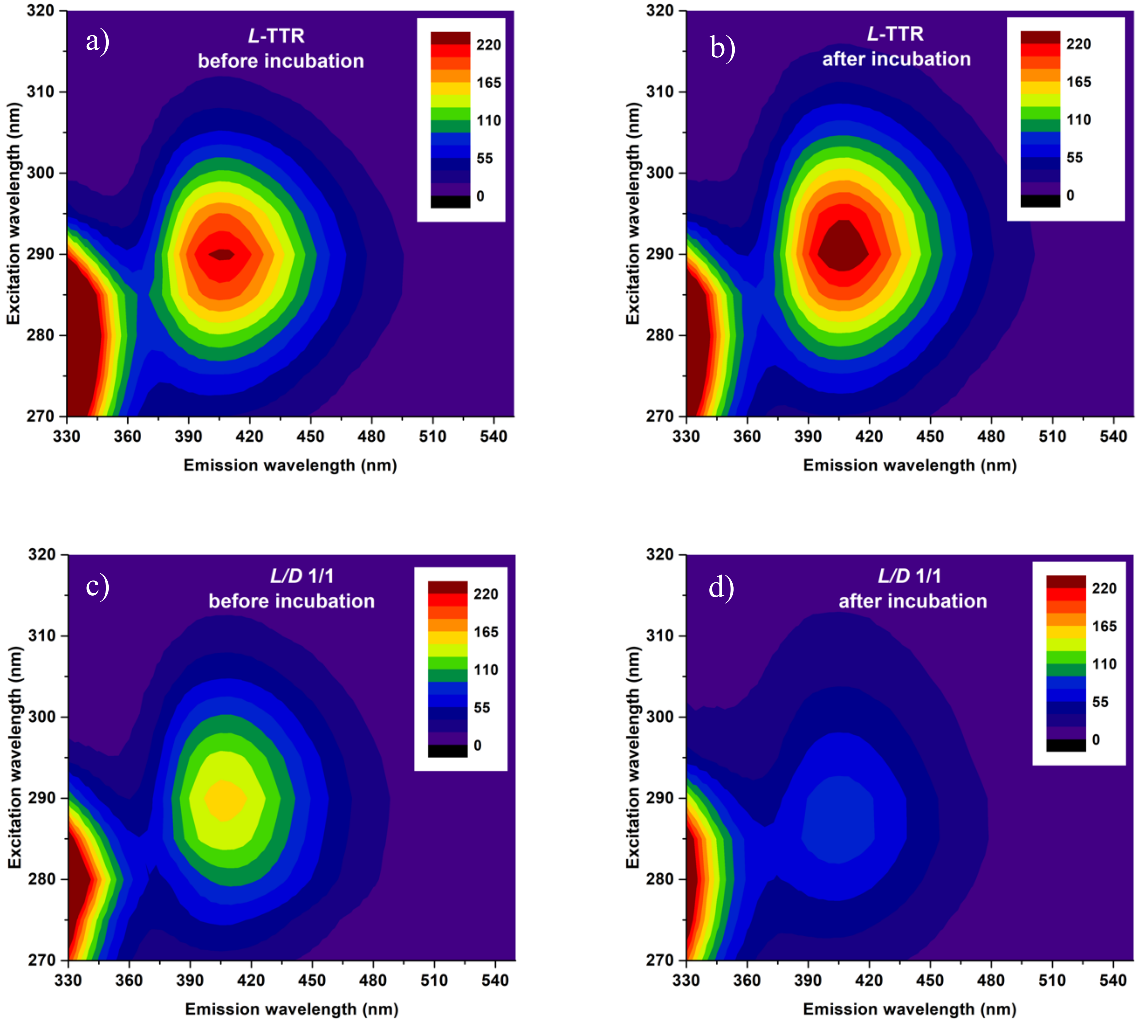

Figure S6. Fluorescence excitation-emission maps in the excitation range 270-320 nm recorded for *L*-TTR and the racemate (*L/D* 1/1) before (a, c) and after incubation (b, d), respectively.

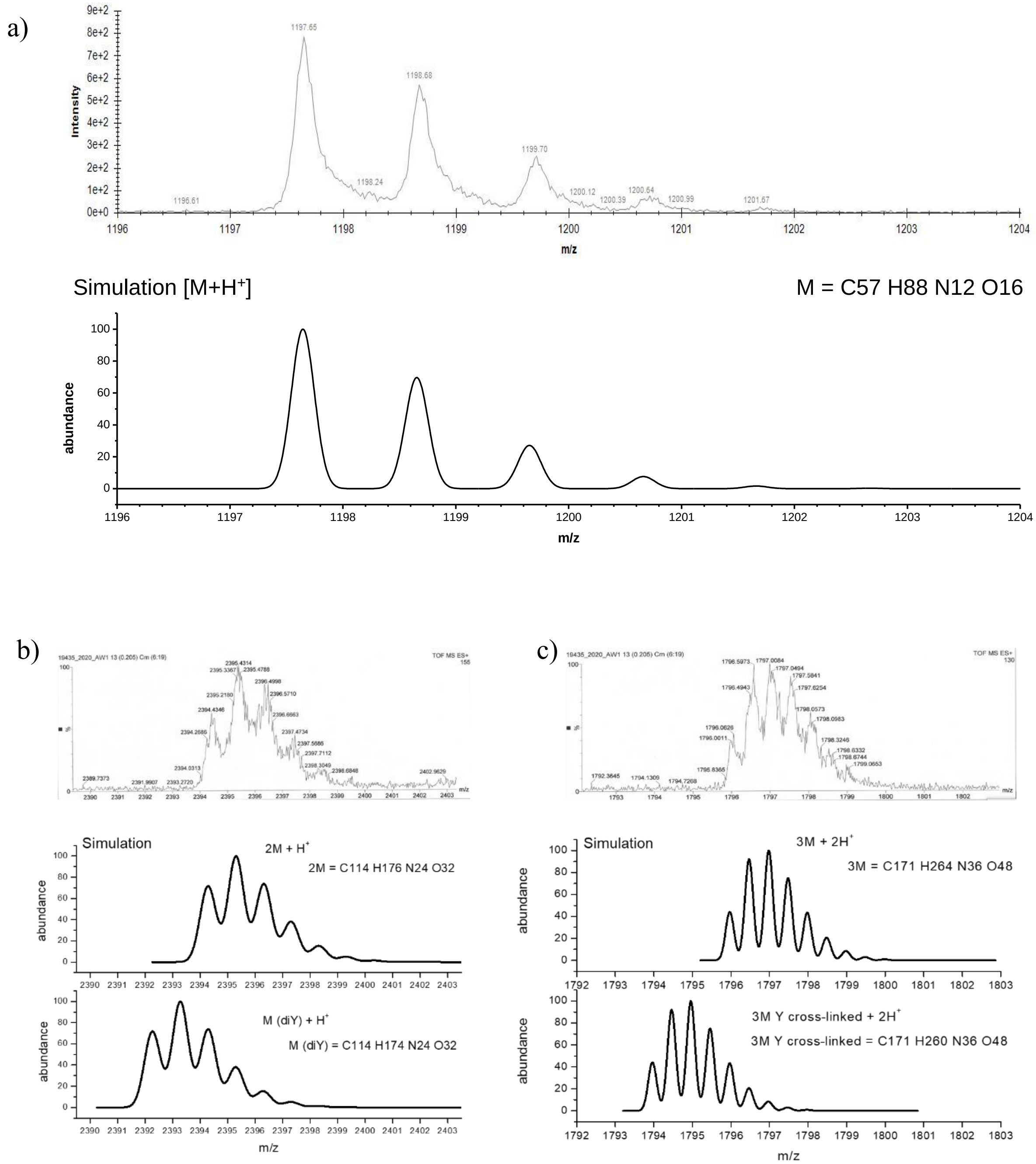

Figure S7. ESI-MS spectra confirming the absence of covalent Tyr cross-links in *L*-TTR(105-115). Experimental spectrum with isotopic resolution for $1H^+$ charge state (top panel) and its theoretical simulation using IsoPro (v.3) software (lower panel) (a). Experimental spectra for ion clusters $2M{+}H^+$ and $3M{+}2H^+$ (top panels) and the corresponding theoretical simulations (middle panels) (b, c). In addition, theoretical simulations were performed for putative Tyr cross-linked species (lower panels) showing that their masses are lower than the observed values. The spectra were acquired and analyzed for *D*-TTR(105-115) and the racemic mixture as well, where the results were similar to shown in this Figure.

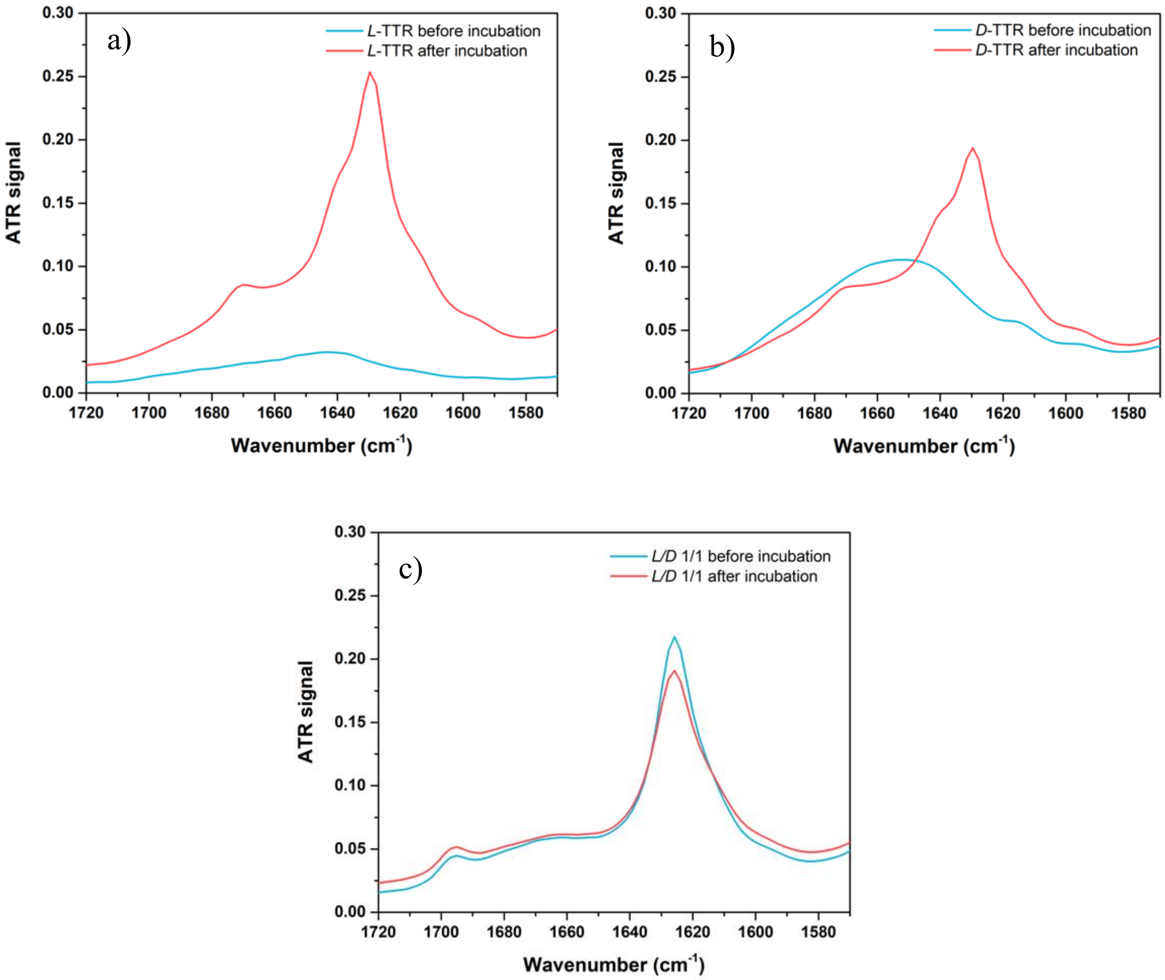

Figure S8. ATR-FTIR spectra of *L*-TTR (a), *D*-TTR (b) and racemic mixture *L*/*D*-TTR (c) before (blue) and after incubation (red).

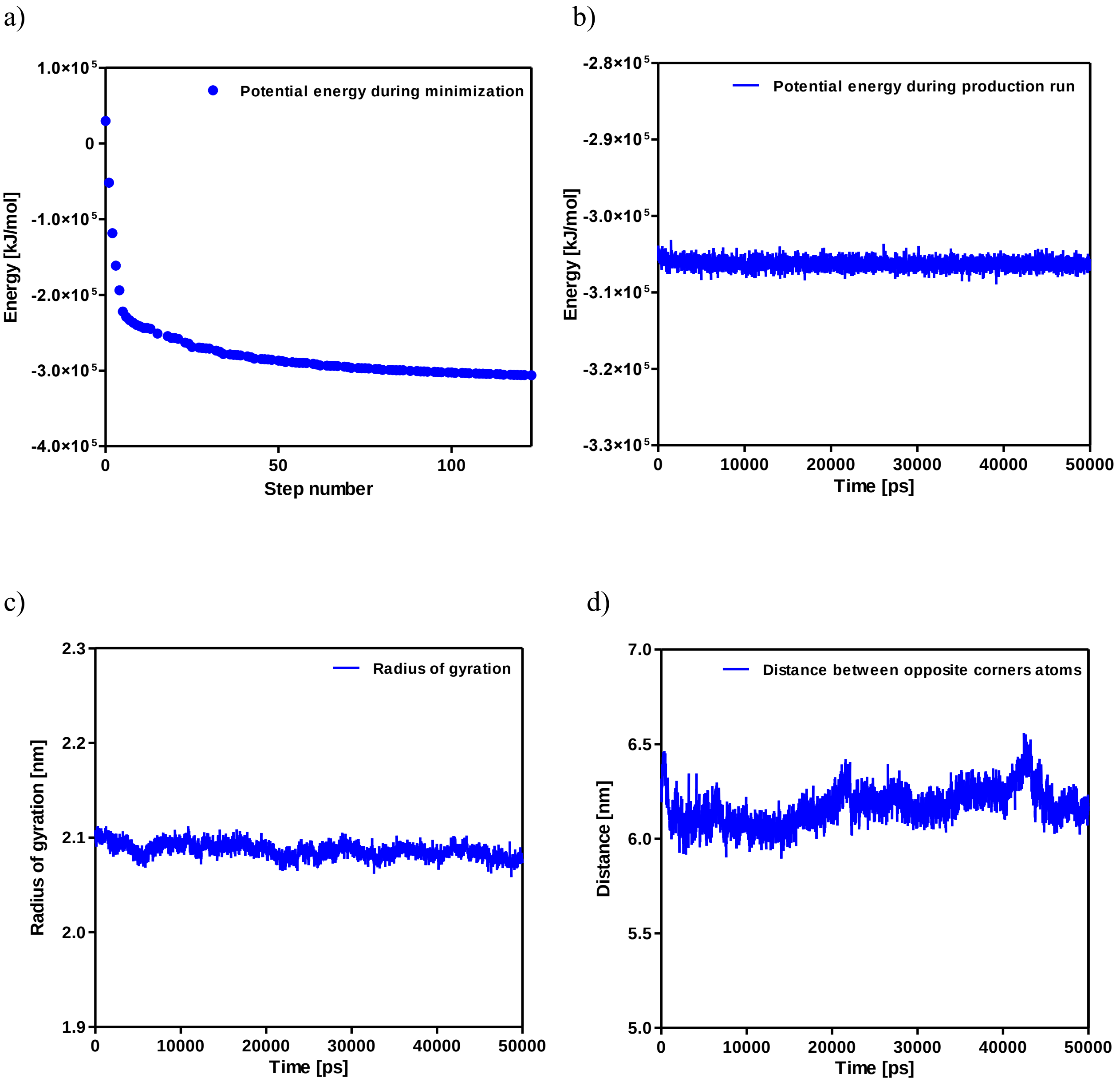

Figure S9. Potential energy during minimization of MD simulation of antiparallel *L*/*D*-TTR fibrils (a), potential energy during production run (b), radius of gyration (c), the distance between the opposite corners atoms (d).

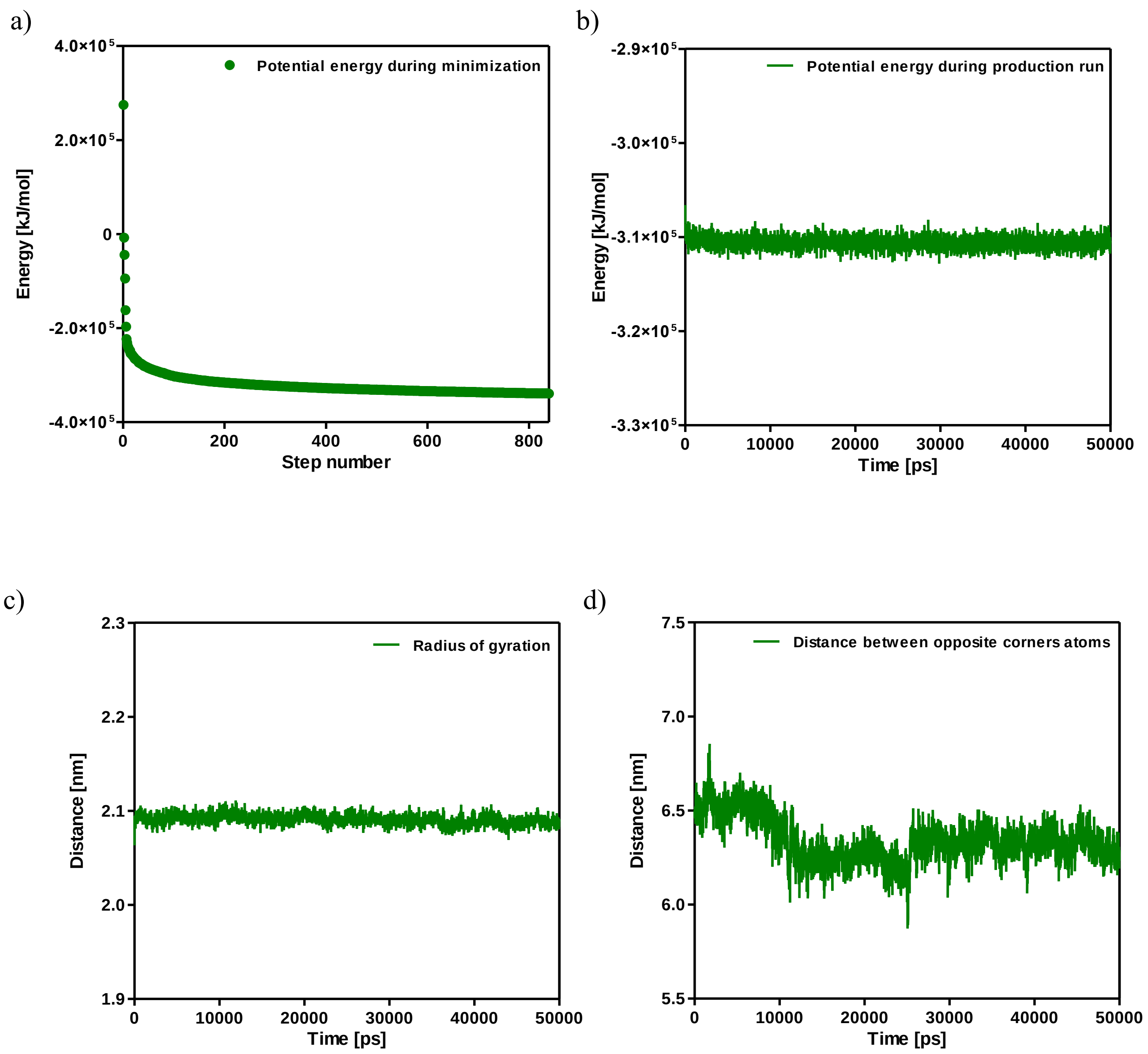

Figure S10. Potential energy during minimization of MD simulation of parallel *L*-TTR fibrils (a), potential energy during production run (b), radius of gyration (c), the distance between the opposite corners atoms (d).

a)

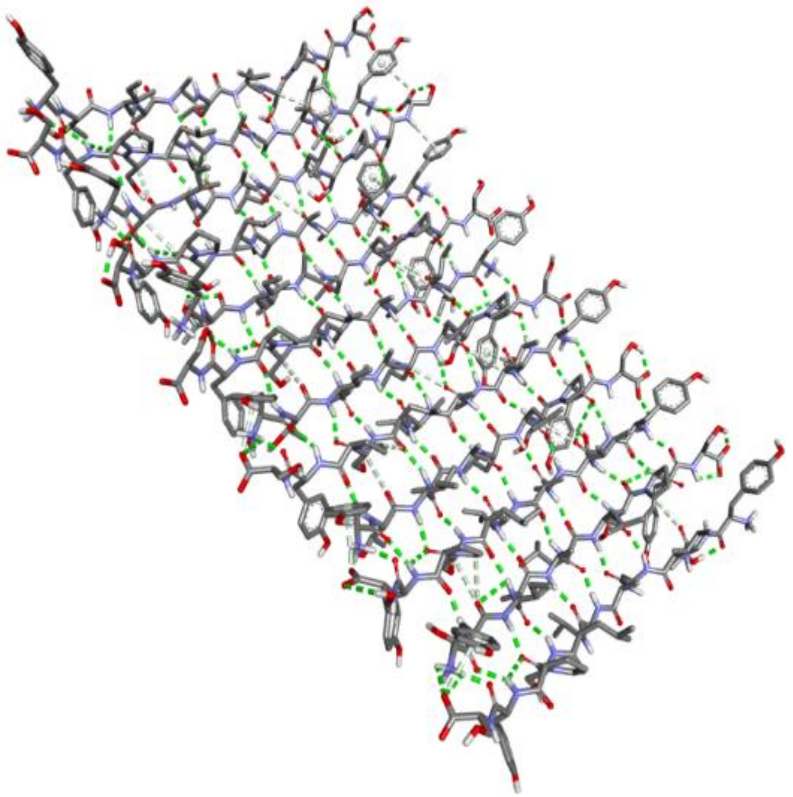

b)

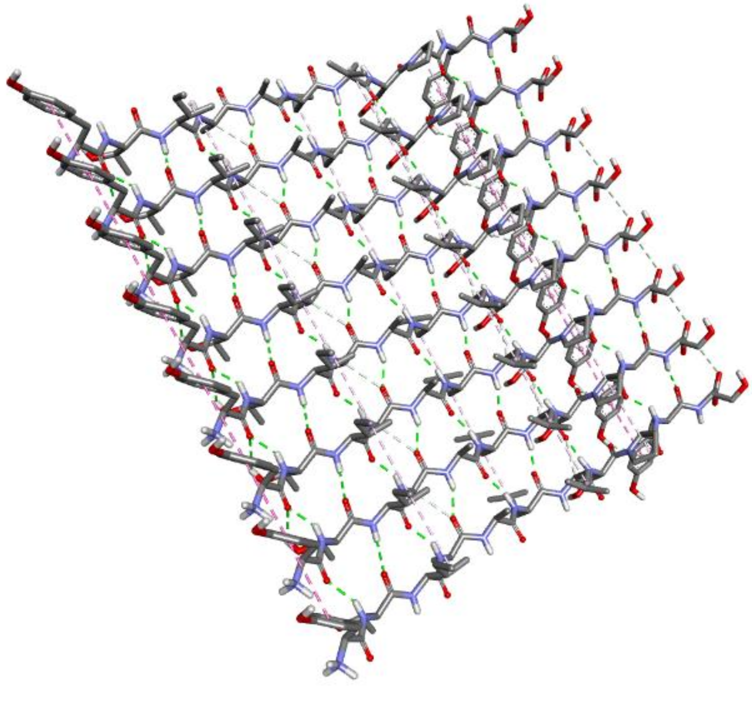

Figure S11. The model structure representing one layer composed of alternating *L*- and *D*-strands in racemic mixture (a) and of adjacent *L*-strands in *L*-TTR(105-115) enantiomer (b).
